# Supplementary material for: Multichannel resonant acoustic rheometry system for quantification of coagulation of multiple human plasma samples
Source: Sci Rep. 2023 Nov 7;13:19237. doi: 10.1038/s41598-023-46518-w (PMC10630367; doi:10.1038/s41598-023-46518-w)
Supplement: Supplementary file 1 — Supplementary Information. [file 41598_2023_46518_MOESM1_ESM.pdf]

Supplemental Information

Multichannel Resonant Acoustic Rheometry System

for Quantification of Coagulation of Multiple Human Plasma Samples

Christina Hendren,[1] Weiping Li,[1] Jan P. Stegemann,[1] Timothy L. Hall,[1,*] and Cheri. X. Deng[1,2,*]

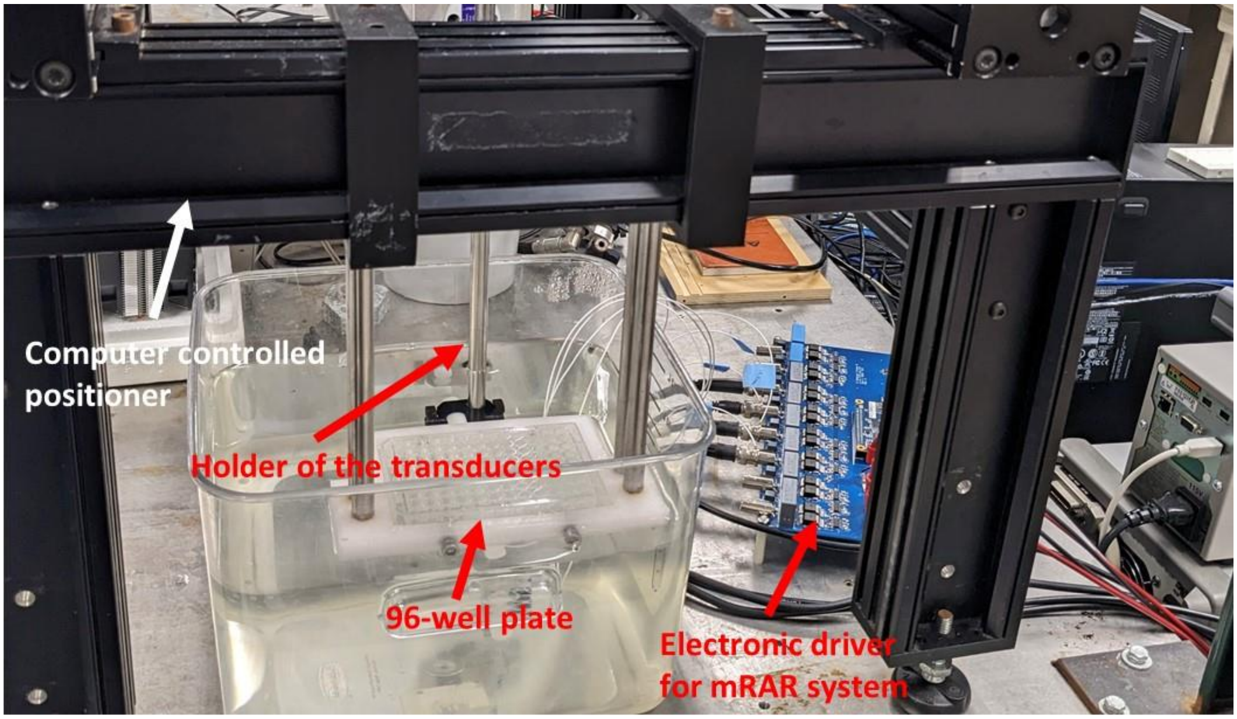

Figure S1. Photograph of the prototype mRAR system. The transducers, hold by a holder mounted on a mechanical positioner, are placed below the 96-well plate.

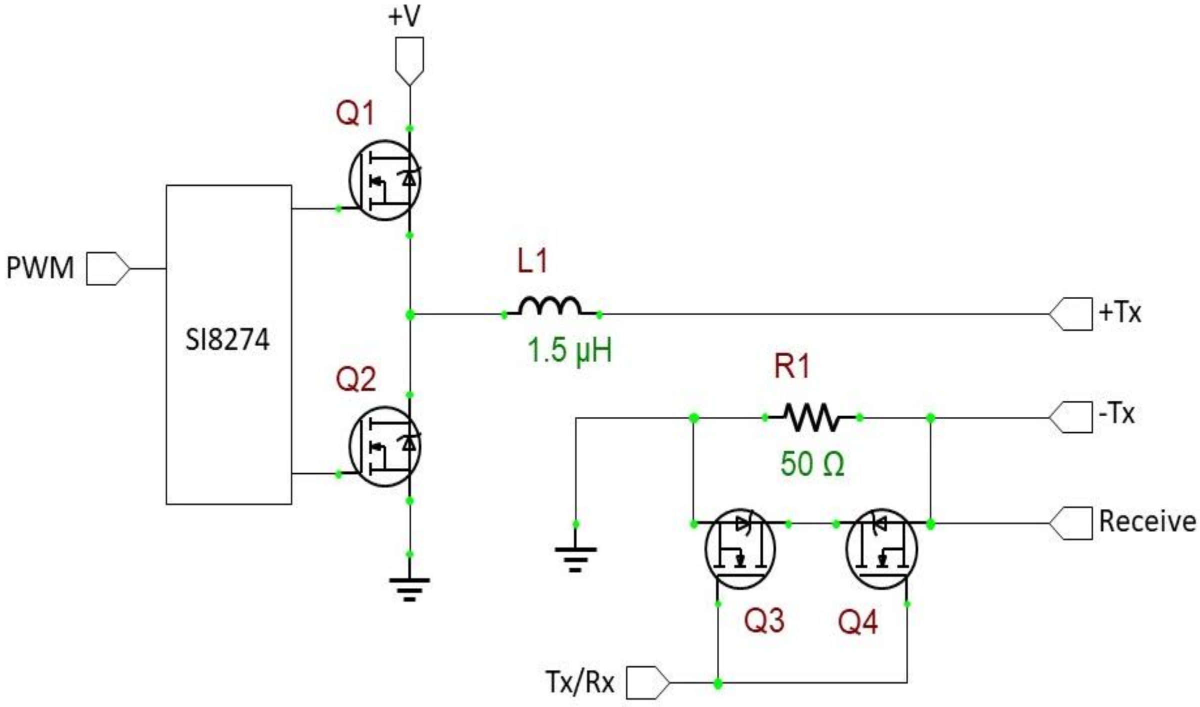

Figure S2. Simplified schematic of the transducer driver/receiver. A standard “push-pull” drive configuration is used for transmit. Received signals are detected by a ground path sense resistor (R1) and digitized by a Picoscope (not pictured) connected at the terminal marked “Receive”. The transducers are driven by a pair of silicon carbide MOSFETs (Q1, Q2) in a “push-pull” configuration controlled by an isolated gate driver (Si8274). A matching inductor L1 converts the resulting square waves into a more sinusoidal shape while boosting the peak voltage. To detect reflections from the target surface, a current sensing scheme was used through sense resistor R1. Another pair of low voltage silicon MOSFETs (Q3, Q4) are turned on to short the sense resistor during transmit and then turned off during receive.

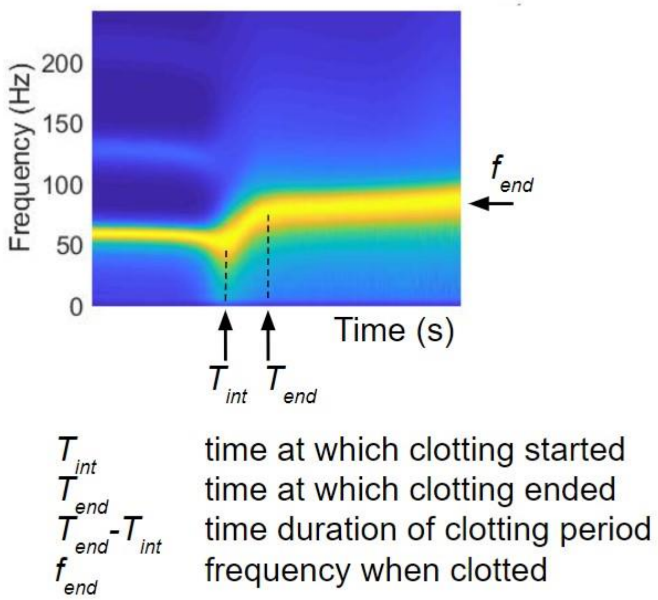

Figure S3. A schmatic showing the parameters derived from the spectrogram measured by RAR including the clotting start time, T_{int}, the clotting end time T_{end}, duration T_{end} -T_{int}, and the final frequency, f_{end}, respectively.

Table S1: Results of p-values for linear regression statistical tests on Final Frequency

	Comparison group					
Reference group	**normal**	**low INR**	**med INR**	**high INR**	**kaolin**	**TF**
**normal TF**		1.42E-09	7.08E-06	1.31E-08	0.75857	
**normal kaolin**		1.11E-15	4.95E-11	2.20E-07		0.75857
**low INR TF**	1.42E-09		0.031378	0.57175	3.26E-05	
**low INR kaolin**	1.11E-15		0.0059047	0.0014688		3.26E-05
**med INR TF**	7.08E-06	0.031378		0.10653	0.00049107	
**med INR kaolin**	4.95E-11	0.0059047		0.36727		0.00049107
**high INR TF**	1.31E-08	0.57175	0.10653		0.38939	
**high INR kaolin**	2.20E-07	0.0014688	0.36727			0.38939

Table S2: Results of p-values for linear regression statistical tests on clotting start time

	Comparison group					
Reference group	**normal**	**low INR**	**med INR**	**high INR**	**kaolin**	**TF**
**normal TF**		6.69E-05	5.10E-25	5.22E-33	6.25E-11	
**normal kaolin**		2.96E-07	8.44E-19	8.00E-23		6.25E-11
**low INR TF**	6.69E-05		6.09E-19	6.20E-28	1.77E-11	
**low INR kaolin**	2.96E-07		4.41E-08	6.48E-15		1.77E-11
**med INR TF**	5.10E-25	6.09E-19		1.65E-08	0.049387	
**med INR kaolin**	8.44E-19	4.41E-08		1.37E-06		0.049387
**high INR TF**	5.22E-33	6.20E-28	1.65E-08		0.048783	
**high INR kaolin**	8.00E-23	6.48E-15	1.37E-06			0.048783

Table S3: Results of p-values for linear regression statistical tests on Clotting Duration

	Comparison group					
Reference group	**normal**	**low INR**	**med INR**	**high INR**	**kaolin**	**TF**
**normal TF**		0.047011	7.37E-14	9.54E-15	0.00050651	
**normal kaolin**		0.0038293	2.18E-12	2.99E-28		0.00050651
**low INR TF**	0.047011		3.61E-10	7.67E-11	0.00012675	
**low INR kaolin**	0.0038293		3.01E-06	5.34E-24		0.00012675
**med INR TF**	7.37E-14	3.61E-10		0.9958	0.044584	
**med INR kaolin**	2.18E-12	3.01E-06		9.78E-19		0.044584
**high INR TF**	9.54E-15	7.67E-11	0.9958		9.56E-22	
**high INR kaolin**	2.99E-28	5.34E-24	9.78E-19			9.56E-22
